# Ultrasonography of the omasum in 30 Saanen goats

**DOI:** 10.1186/1746-6148-7-11

**Published:** 2011-02-21

**Authors:** Ueli Braun, Désirée Jacquat

**Affiliations:** 1 Department of Farm Animals, Vetsuisse Faculty, University of Zurich, Zurich, Switzerland

## Abstract

**Background:**

Primary diseases of the omasum are uncommon in goats, although the omasum may be involved in various gastrointestinal disorders. Examination of the caprine omasum via ultrasonography requires a good understanding of the normal appearance of the organ. However, in contrast to cattle, there is a lack of reference information on this topic in goats. Thus, the goal of the present study was to describe the results of ultrasonography of the omasum in 30 healthy Saanen goats.

**Results:**

Ultrasonography was carried out in standing, non-sedated goats using a 5.0 MHz linear transducer. The location and size of the omasum, thickness of the omasal wall and visualisation of the abomasal laminae, contents and contractions were assessed. The omasum was visible from the 9th intercostal space (ICS) in all the goats, and from the 8th and 10th ICSs in 29 and 24 goats, respectively. The omasum was seen medial to the liver, but only the omasal wall closest to the transducer was visible. The dorsal omasal limit formed a dorsally convex curve running from cranioventral to caudodorsal and was furthest from the dorsal midline in the 6th ICS. The ventral omasal limit formed a ventrally convex curve. The size of the omasum was largest (10.2 ± 3.1 cm) in the 9th ICS and decreased cranially and caudally from this position. Active omasal motility was recorded in 20 goats with 0.3 to 2.0 contractions per minute.

**Conclusions:**

The findings of this study provide reference ranges for the interpretation of the location and size of the omasum in goats with suspected omasal abnormalities. Ultrasonography is an ideal diagnostic tool for evaluation of the omasum, which is not accessible to conventional examination techniques, such as inspection, palpation, percussion and auscultation.

## Background

The caprine omasum is located in the ventral aspect of the cranial part of the abdomen between the rumen and liver [1]. The omasum has 80 to 100 laminae, which vary in size and serve to absorb water, minerals and short-chain fatty acids [2]. Although the omasum is involved in various gastrointestinal disorders, primary diseases of the omasum are uncommon and occur chiefly in cattle [3,4]. They include omasal impaction and inflammation as well as paresis, which is characterised by cessation of influx of ingesta from the reticulum leading to anterior functional stenosis. Peritonitis may result in secondary inflammatory changes of the omasum, and retrograde flow of ingesta into the omasum may occur in cattle with ileus. Clinical examination of the omasum including palpation, percussion and auscultation are limited because of its location in the cranial abdomen. Haematological evaluation and examination of rumen fluid do not provide specific diagnostic information, and radiography of the omasum is limited, although assessment of the organ is possible using a combination of contrast radiography and artificial pneumoperitoneum [5]. Computed tomography (CT) is an ideal method for examination of the omasum in goats [6]. The characteristic ovoid shape of the omasum allows it to be easily differentiated from neighbouring organs. On CT images, the abomasum is round in transverse section and has a typical pattern of alternating light grey laminae and hypodense ingesta. However, computed tomography is expensive and requires general anaesthesia of the patient. Ultrasonography on the other hand provides a practical and straightforward method for the examination of the omasum in healthy cows [7] and in cows with various gastrointestinal diseases [8]. A recent report described the ultrasonographic appearance of the omasum in a goat with mesothelioma [9]. Although nodular changes were readily detected in the omasum, the overall assessment of the organ was hampered by a lack of reference values for the ultrasonographic appearance, location and size of the caprine omasum. The goal of the present study was to determine the appearance, location and size of the omasum via ultrasonography in healthy female goats.

## Methods

### Animals

Thirty clinically healthy, non-lactating, female, Saanen goats were used. They ranged in age from 2.5 to 6.5 years (mean ± SD = 4.9 ± 1.10 years) and weighed 42 to 86 kg (mean, 61.9 kg). The goats originated from two farms and had been sold for slaughter. All the goats were deemed healthy based on the results of a thorough clinical examination, a complete blood cell count, biochemical profile, urinalysis, and examination of rumen juice and faeces; the results have been described in detail [10]. The goats were housed in two large pens, which were bedded with straw daily. They were fed hay until the time of ultrasonographic examination. The study protocol was approved by the Animal Care Committee of the Canton of Zurich, Switzerland.

### Ultrasonography of the omasum

**Figure 1:**
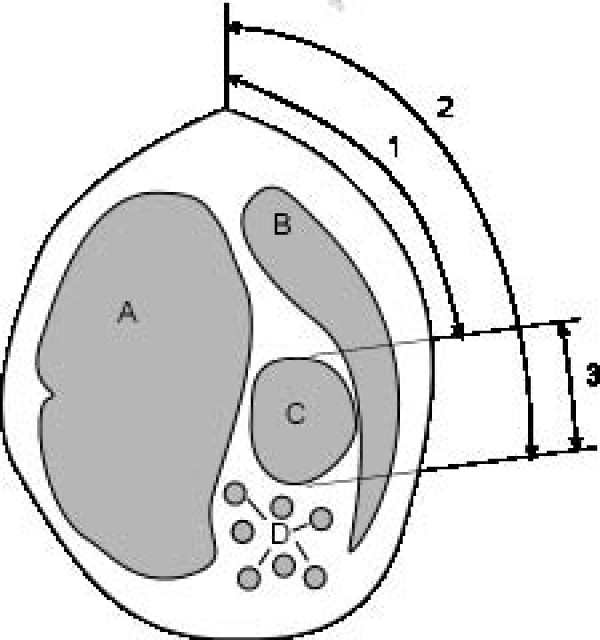
**Position and size of the omasum**. Schematic representation of determination of the position and size of the omasum. A Rumen, B Liver, C Omasum, D Small intestines, 1 Distance between dorsal limit of omasum and dorsal midline, 2 Distance between ventral limit of omasum and dorsal midline, 3 Size of omasum.

**Figure 2:**
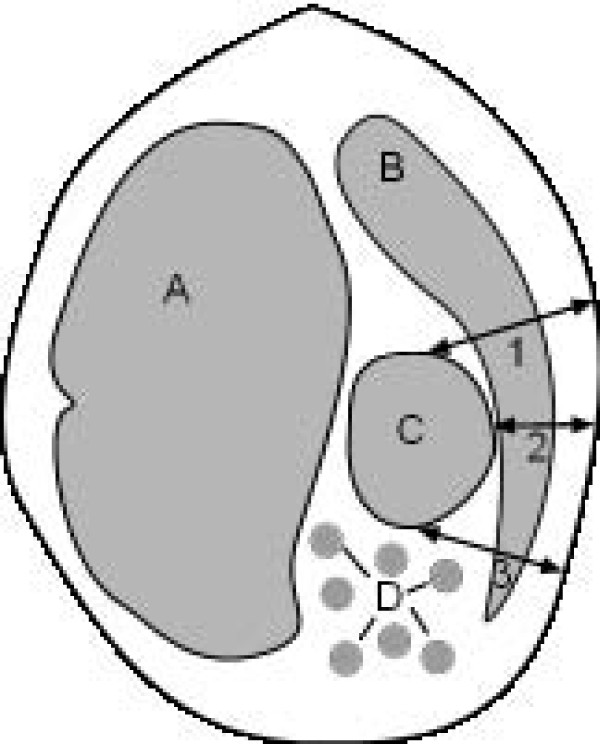
**Distance of the omasum from the abdominal wall**. Schematic representation of determination of the distance of the omasum from the abdominal wall. A Rumen, B Liver, C Omasum, D Small intestines, 1 Distance between dorsal limit of omasum and abdominal wall, 2 Smallest distance between omasum and abdominal wall, 3 Distance between ventral limit of omasum and abdominal wall.

Ultrasonographic examinations were carried out in standing, non-sedated animals as described previously [11] using a 5.0 MHz linear transducer with a penetration depth of 10 cm (EUB 8500, Hitachi Medical Systems, Zug, Switzerland). The hair was clipped on the right thoracic wall from the caudal border of the shoulder to just caudal to the last rib and from the dorsal midline to the ventral midline. All intercostal spaces (ICSs) on the right were examined from caudal to cranial and from dorsal to ventral with the transducer held parallel to the ribs and perpendicular to the omasum to assess the omasal wall, laminae, contents and contractions. The thickness of the omasal wall was measured electronically using the cursors on the monitor. To determine the location and size of the omasum, the distance between the dorsal limit of the omasum and the dorsal midline (distance 1) and the distance between the ventral limit of the abomasum and dorsal midline (distance 2) were measured with a tape measure (Figure 1). The size of the omasum was determined by subtracting distance 1 from distance 2. The distance of the omasum from the abdominal wall was determined by measuring the distances between the dorsal and ventral limits of the omasum and the peritoneum of the abdominal wall, and between the closest part of the omasum and the peritoneum during maximum inspiration in each ICS (Figure 2). This was done electronically on frozen images using the cursors on the monitor. Abomasal motility was assessed by ultrasound video-recordings, which were 5 minutes in length. The total length of time required for ultrasonographic examination of the omasum was 15 to 20 minutes.

### Postmortem examination

The goats were slaughtered (n = 14) or euthanased (n = 16) after the ultrasonographic examination. The omasum was examined macroscopically in the slaughtered goats. The euthanased goats, which were also used in other studies [6,10,12], were frozen and cut into 1.0 to 1.5 cm-thick transverse sections. The omasum was examined on these sections to determine abnormalities and to verify visual observations made via ultrasonography.

### Statistical analysis

Frequencies, means and standard deviations were calculated using the statistical software program StatView 5.1 (SAS Institute, Cary, USA).

## Results

### Ultrasonographic appearance

**Figure 3:**
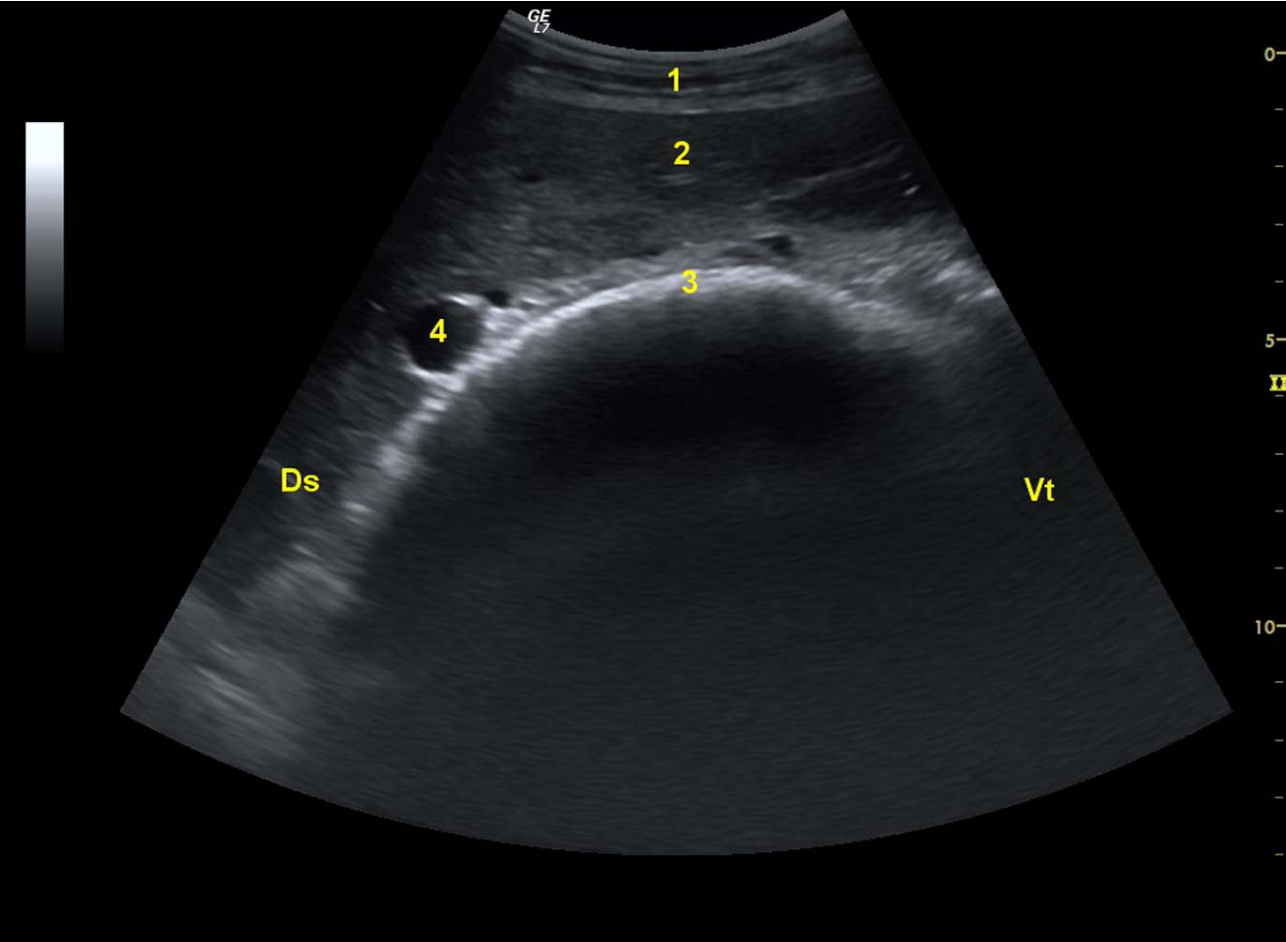
**Ultrasonogram of the omasum**. Ultrasonogram of the omasum and liver of a 5-year-old Saanen goat viewed from the 9th intercostal space with a 5.0-MHz convex transducer. The omasum appears as a curved echogenic line medial to the liver. 1 Abdominal wall, 2 Liver, 3 Omasum, 4 Portal vein, Ds Dorsal, Vt Ventral.

Starting dorsally and progressing ventrally, the back musculature, lungs, liver and omasum were seen in the 6th to 11th ICSs on the right side (Figure 3). The omasum was located medial to the liver. In contrast to cattle, the omasum was never located immediately adjacent to the abdominal wall in goats. The various layers of the abdominal wall appeared as bands of varying echogenicity. The ultrasonographic appearance of the lungs, liver and omasum was analogous to that described in cows [13,14]. The omasal wall closest to the transducer was crescent-shaped and located medial to the liver. The omasal folds and remainder of the wall could not be visualised because of gas within the omasum. The echogenic tunica mucosa and tela submucosa of the omasal wall were clearly visible in all the goats. The mean thickness of the omasal wall was 4.3 ± 1.6 mm.

### Visibility and size of the omasum in the 6th to 11th ICS

**Figure 4:**
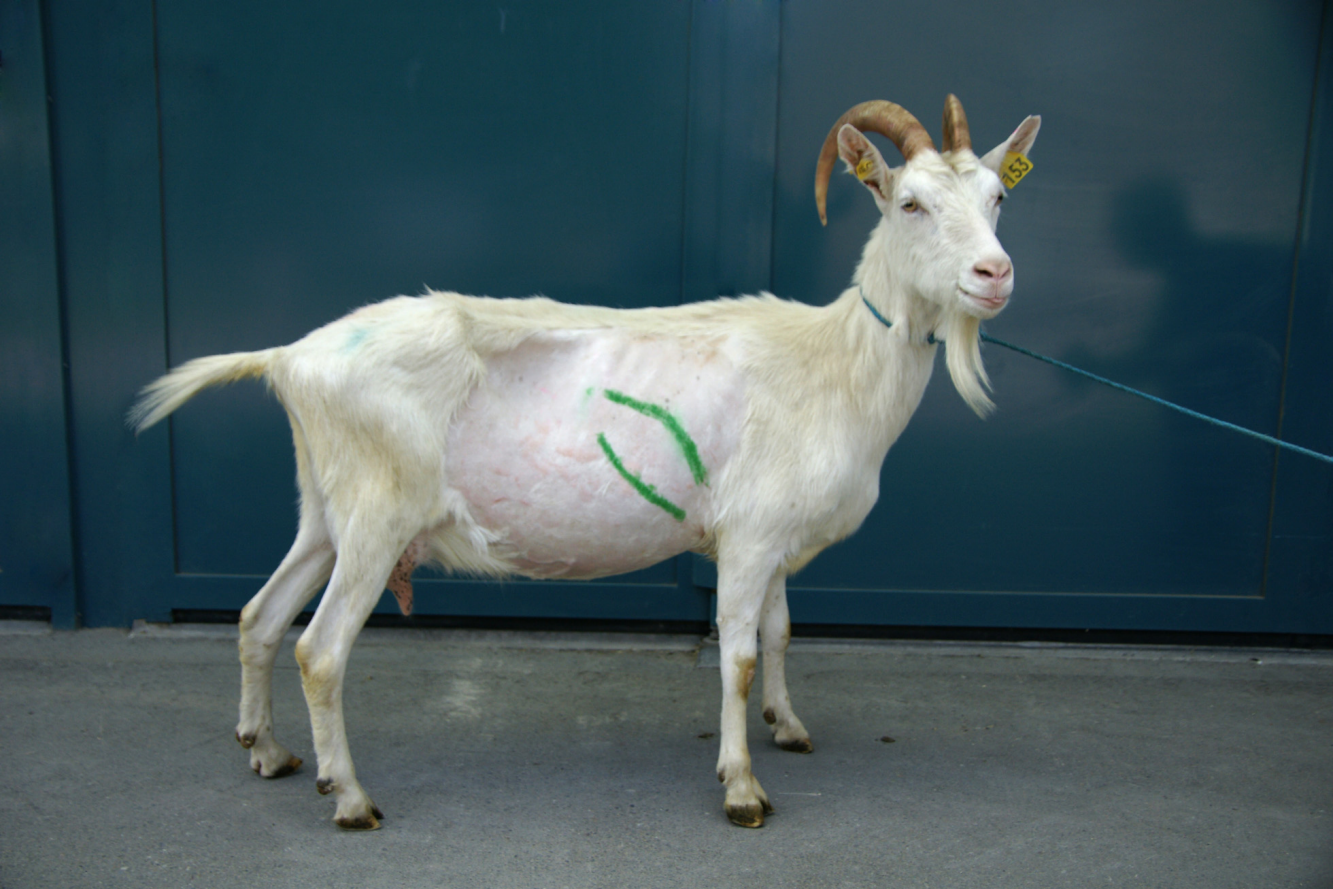
**Location of the omasum**. The lines drawn on the goat represent the dorsal and ventral limits of the omasum from the 6th to the 11th intercostal space. These limits correspond to the mean positions of the dorsal and ventral limits of the omasum in 30 healthy goats.

The omasum was visible in the 9th ICS in all the goats, in the 8th ICS in 29, in the 10th ICS in 24, in the 7th ICS in 16, in the 6th ICS in four and in the 11th ICS in three goats (Table 1). The omasum was seen in three consecutive ICSs in 17 goats, in four consecutive ICSs in nine goats and in five consecutive ICS in four goats. The dorsal limit of the omasum formed a dorsally convex curve running from cranioventral to caudodorsal (Figure 4). The maximum distance between the dorsal limit of the omasum and the dorsal midline was measured in the 6th ICS and was 25.9 ± 2.4 cm. This distance progressively decreased caudally and was smallest (6.5 ± 4.4 cm) in the 11th ICS. The ventral limit of the omasum formed a ventrally convex curve; the distance between the ventral limit of the omasum and the dorsal midline was 33.6 ± 2.9 cm in the 6th ICS and 22.7 ± 4.7 cm in the 11th ICS. The maximum size of the omasum was 10.2 ± 3.1 cm measured in the 9th ICS and the minimum size was 6.7 ± 1.3 cm measured in the 11th ICS.

**Table 1:** Results of ultrasonographic examination of the omasum of 30 healthy Saanen goats.

Variable	ICS (No. of goats)	Mean	SD	Range
Dorsal visible limit*	6 (4)	25.9	2.4	24.0 - 29.0
	
	7 (16)	23.2	2.7	20.0 - 29.0
	
	8 (29)	20.1	2.6	16.0 - 28.0
	
	9 (30)	17.8	2.5	13.0 - 24.0
	
	10 (24)	16.9	3.5	10.0 - 25.0
	
	11 (3)	16.5	4.4	1.5 - 20.0

Ventral visible limit*	6 (5)	33.6	2.9	29.0 - 36.0
	
	7 (16)	30.8	5.1	23.5 - 41.0
	
	8 (29)	29.0	3.8	23.0 - 39.0
	
	9 (30)	27.9	3.5	22.0 - 37.0
	
	10 (23)	25.5	4.6	18.0 - 36.0
	
	11 (3)	22.7	4.7	17.5 - 26.5

Size (cm)	6 (4)	8.9	2.6	6.5 - 12.0
	
	7 (16)	7.1	4.8	3.0 - 21.0
	
	8 (29)	9.2	3.3	5.0 - 18.0
	
	9 (30)	10.2	3.1	5.0 - 20.0
	
	10 (24)	8.5	2.6	5.0 - 15.0
	
	11 (4)	6.7	1.3	6.0 - 9.0

The distances listed are defined in Figure 1.

ICS = Intercostal space

* Centimetres from the dorsal midline

### Distance between the omasum and abdominal wall

The distance between the dorsal limit of the omasum and the abdominal wall ranged from 4.7 to 6.7 cm, and the distance between the ventral limit of the omasum and the abdominal ranged from 5.8 to 6.1 cm (Table 2). The distance between the omasum and the abdominal wall was smallest (2.4 cm) in the 6th ICS.

**Table 2:** Distance between the visible dorsal and ventral limits of the omasum and abdominal wall and between the abdominal wall and the part of the omasum closest to the abdominal wall in 30 healthy Saanen goats.

	Distance between omasum and abdominal wall (cm, mean ± SD)		
	
ICS (No. of goats)	Dorsal limit of omasum	Part of omasum closest to abdominal wall	Ventral limit of omasum
6 (4)	4.7 ± 0.6	2.4 ± 0.8	NE

7 (16)	5.6 ± 1.0	4.0 ± 1.3	5.8 ± 0.9

8 (29)	6.2 ± 1.1	4.1 ± 0.9	5.9 ± 1.0

9 (30)	6.7 ± 1.3	4.2 ± 1.3	6.1 ± 1.2

10 (24)	6.5 ± 1.4	4.4 ± 1.4	6.1 ± 1.1

11 (4)	6.4 ± 1.1	3.8 ± 0.6	6.1 ± 1.0

The distances are defined in Figure 2.

ICS = Intercostal space

NE Not examined

### Adjacent organs

The lungs and liver were seen dorsal to the omasum in the 6th to 11th ICS (Table 3). The reticulum or abomasum and further caudally the small intestines were seen ventrally. The lungs, reticulum and liver were seen cranial to the omasum, and the small intestines and sometimes the liver were seen caudal to the omasum. The liver, gallbladder and small intestines were seen lateral to the omasum. No organs were seen medial to the omasum.

**Table 3:** Organs seen dorsal, ventral, cranial, caudal and lateral to the omasum on ultrasonograms in 30 healthy Saanen goats.

Location	Neighbouring organs	Intercostal spaces (number of goats)					
		
		6 (n = 4)	7 (n = 16)	8 (n = 29)	9 (n = 30)	10 (n = 24)	11 (n = 3)
Dorsal	Lung	4	15	27	28	19	3
	
	Liver	0	0	4	4	6	1

Ventral	Reticulum	4	16	19	4	0	0
	
	Abomasum	0	0	10	19	7	0
	
	Intestines	0	0	0	9	16	4

Cranial	Lung	1	3	3	0	0	0
	
	Reticulum	3	9	10	1	0	0
	
	Liver	0	1	3	1	0	0

Caudal	Intestines	0	0	0	4	21	3
	
	Liver	0	0	0	1	1	0

Lateral	Liver	4	16	29	30	23	2
	
	Gallbladder	0	1	4	7	1	0
	
	Intestines	0	0	0	0	5	1

### Omasal motility

**Figure 5:**
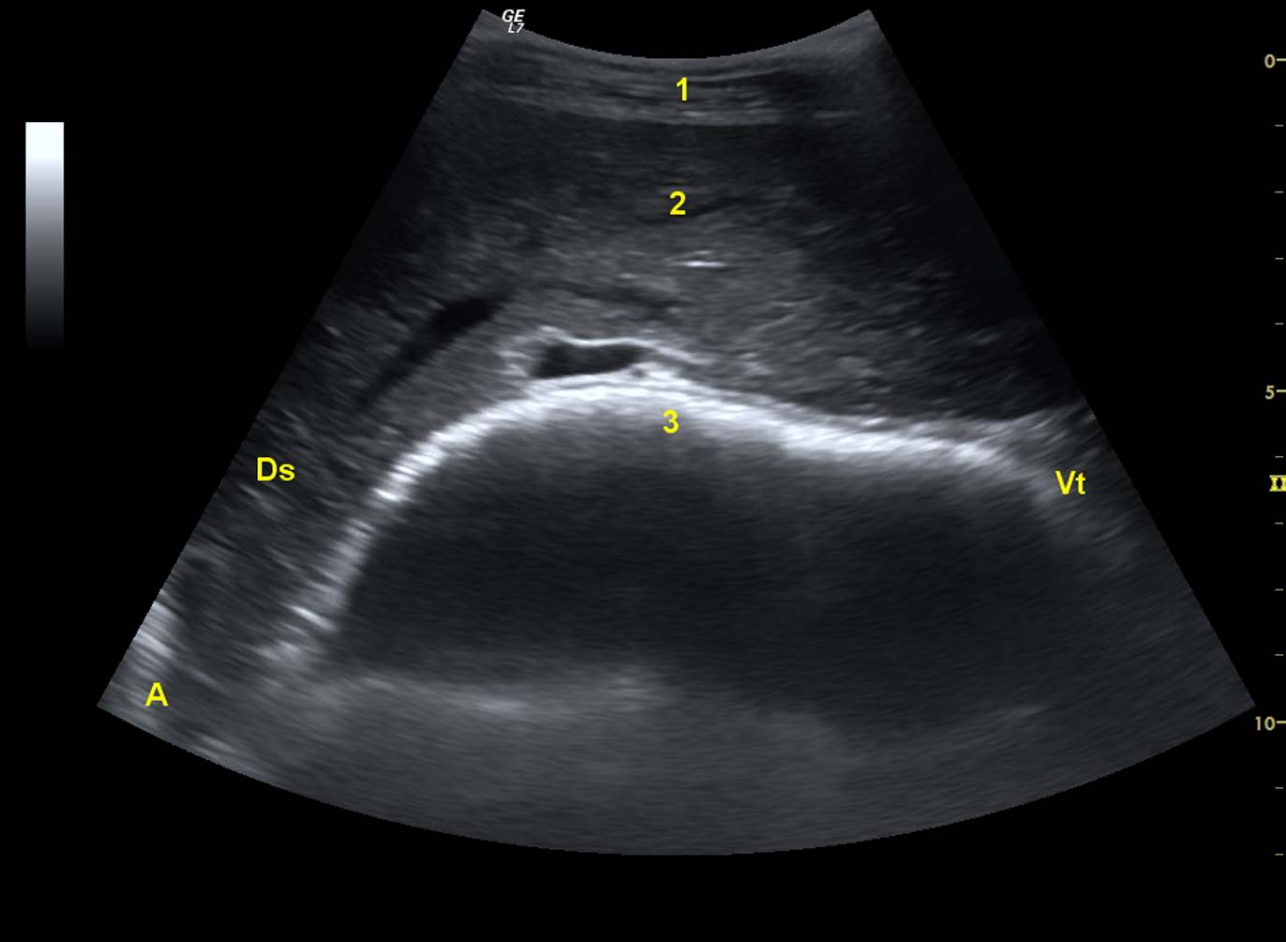
**Omasum during contraction**. Ultrasonogram of the omasum and liver of a 3.5-year-old Saanen goat before contraction of the omasum. 1 Abdominal wall, 2 Liver, 3 Omasum, 4 Portal vein, Ds Dorsal, Vt Ventral.

**Figure 6:**
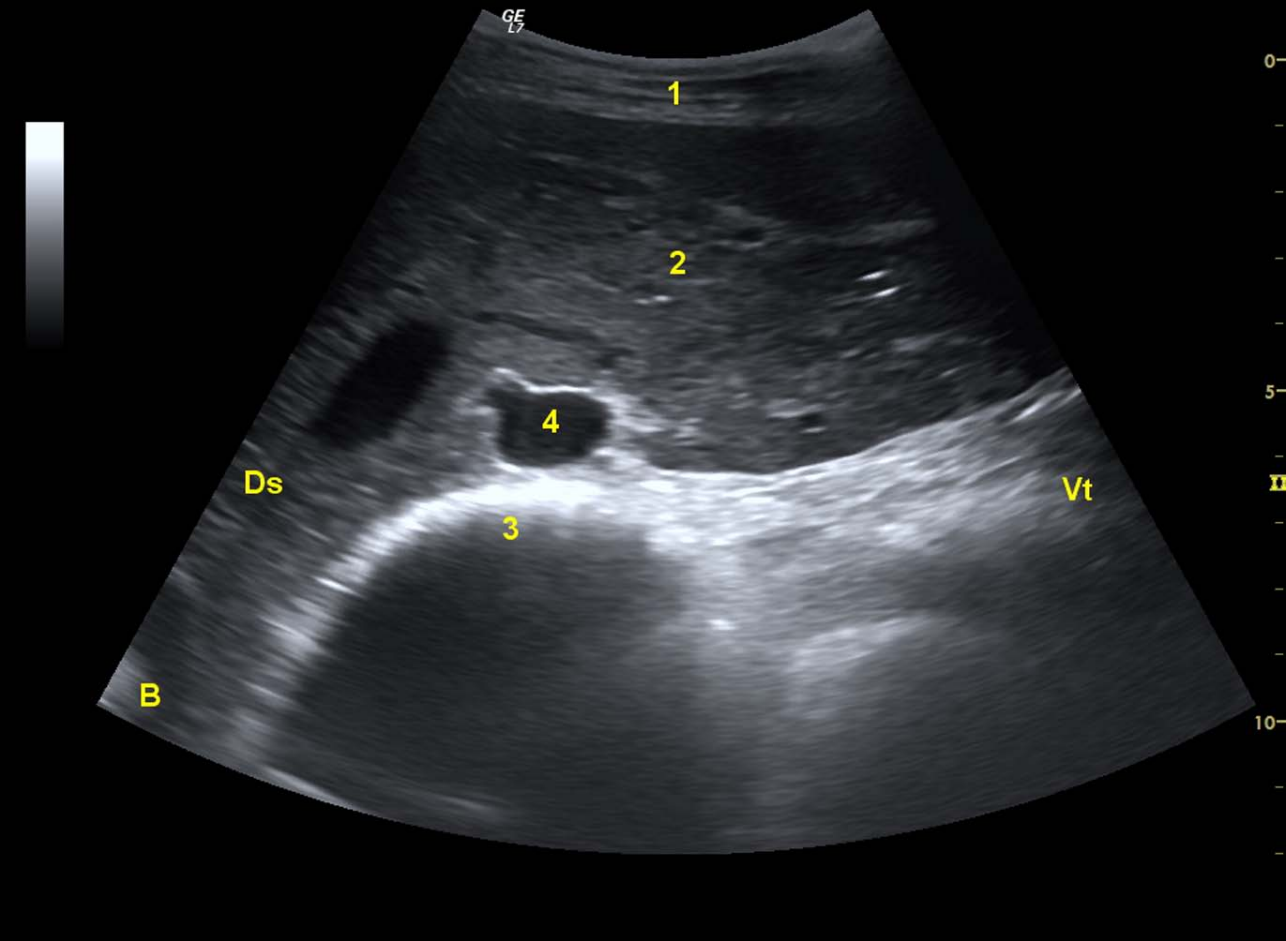
**Omasum during contraction**. Ultrasonogram of the omasum and liver of a 3.5-year-old Saanen goat during contraction of the omasum. Key see Figure 5.

**Figure 7:**
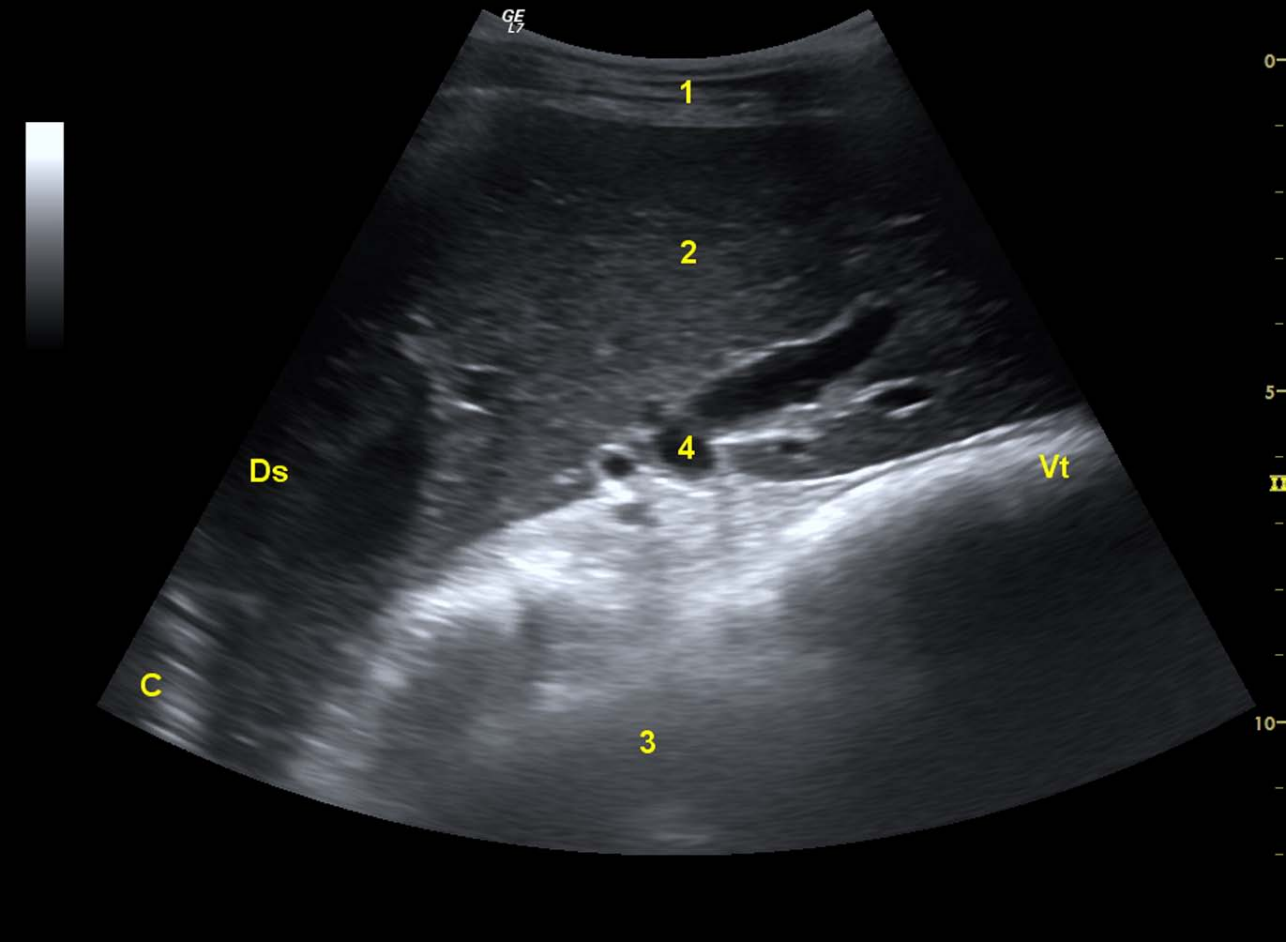
**Omasum during contraction**. Ultrasonogram of the omasum and liver of a 3.5-year-old Saanen goat at the point of maximum contraction of the omasum. Key see Figure 5.

There were regular omasal movements synchronous with respiratory movements. Active omasal motility was seen in 20 goats, in which the omasum contracted by several centimetres and then returned to its original size (Figures 5, 6 and 7). Strong omasal contractions that resulted in the omasum disappearing from the ultrasound monitor occurred in two goats. The number of contractions varied from 0.3 to 2.0 (1.10 ± 0.4) per minute, and each contraction lasted 4.5 to 9.2 s (6.4 ± 1.1 s). The interval between two contractions ranged from 28 to 52 s (40.4 ± 10.4 s).

### Postmortem examination

Postmortem examination of the omasum revealed no abnormal findings.

## Discussion

The omasum was visible from the 9th ICS in all the goats and was considered the optimal location for scanning the organ. The omasum was also visible from the 8th ICS in all but one goat, and from the 10th ICS in 24. The ultrasonographic appearance of the caprine omasum was similar to that of the cow; only the omasal wall closest to the transducer was visible because of the gaseous nature of the omasal contents. The inability to image the distal wall of the omasum was not attributable to the ultrasound machine because even with a "high end" machine, the distal wall could not be seen. The omasal laminae were not seen in any of the goats. In contrast, the omasal laminae could be clearly visualised in the ovoid to spherical omasum of the buffalo [15], and in cows, the omasal laminae were sometimes seen as short echogenic cone-shaped structures protruding from the mucosal surface [7]. The laminae would probably be seen in goats with liquid omasal contents because in cattle with abomasal reflux [7], the laminae appear as fine echogenic lines running parallel to each other. Although the omasum of the goat was much smaller than that of cattle (mean, 10.2 cm versus 56.9 cm), the maximum size in both species was observed in the 9th ICS, and the size decreased as the transducer was moved either cranially or caudally. However, the contour of the dorsal and ventral limits of the omasum differed between the two species. In goats, both limits ran from cranial to caudal in a slightly curved line and in a caudodorsal direction. In cattle, the dorsal limit was visible as the upper half of a circle and the lower limit as the lower half of a circle [7]. Thus, the omasum of the goat did not appear as spherical as the bovine omasum. The caudodorsal orientation of the omasal limits in goats may be due to a slight forward tipping of the organ, which causes the caudal aspect to be closer to the dorsal midline and the cranial aspect to be closer to the floor of the abdomen [11]. There are pronounced differences between cattle and goats with respect to the distance between the omasum and abdominal wall. In cattle, the omasum is located immediately adjacent to the abdominal wall in the 8th and 9th ICSs, whereas in goats, the omasum was always medial to the liver. The shortest distance between the omasum and abdominal wall was 2.4 cm, which was measured in the 6th ICS, where the thickness of the liver is less than further caudally [12]. The lack of direct contact between the omasum and abdominal wall was also documented in a radiographic contrast study [5]. This finding is clinically significant because it shows that the omasum is not directly accessible from the right abdominal wall.

Active motility of the omasum, in which the size of the organ temporarily decreased by several centimetres, was documented in 20 of the 30 goats. In two goats, the contractions were so strong that the organ briefly moved beyond the penetration capacity of the probe. Active omasal motility was not recorded in healthy cows [7] but was evident in three cows with reticulo-omasal stenosis [8]. In healthy buffalos, in which active omasal motility was distinct, the omasum appeared large and close to the abdominal wall at the start of a contraction, and then retracted from the abdominal wall and became progressively smaller [15], analogous to observations of the present study. This indicates that the contractions were in a mediolateral direction. The frequency of omasal contractions (1.1/min) was similar to the frequency of reticular contractions in goats (1.4/min) [11]. The duration of an omasal contraction (6.4 s) was similar to the duration of a biphasic reticular contraction (6.6 s) but considerably longer than a monophasic reticular contraction [11]. Based on the findings of the present study, it is not possible to determine whether there is an association between omasal motility and omasal health. Future studies are necessary to evaluate reticular and omasal motility simultaneously with two different ultrasound machines to measure the time interval between reticular and omasal motility and to provide further insight into the relationship between the reticulum and omasum.

## Conclusions

The findings of this study provide reference ranges for the interpretation of the location and size of the omasum in goats with suspected omasal abnormalities. Ultrasonography is an important diagnostic tool for evaluation of the omasum because it is not accessible to conventional examination techniques, such as inspection, palpation, percussion and auscultation. The position and size of the omasum can be accurately assessed using ultrasonography. Because omasal diseases in small ruminants have been difficult to diagnose in the past, it is conceivable that they also have been underdiagnosed. Ultrasonography may provide a means of detecting omasal disease more often in small ruminants.

## Competing interests

The authors declare that they have no competing interests.

## Authors' contributions

UB initiated and planned the study, he wrote the manuscript and made the figures. DJ carried out the ultrasonographic examinations under supervision of UB. Both authors have read and approved the manuscript.

## Source of fundings

This study was financed by the University of Zurich, Switzerland.

## References

[B1] Schummer A Wilkens H Nickel R, Schummer A, Seiferle E Rumpfdarm der Wiederkäuer Lehrbuch der Anatomie der Haustiere Band II 1987 Berlin, Paul Parey 158 194

[B2] Martens H von Engelhardt W, Breves G Transportmechanismen im Psalter Physiologie der Haustiere 2004 Stuttgart, Enke-Verlag 362 363

[B3] Dirksen G Dirksen G, Gründer HD, Stöber M Krankheiten des Psalters Innere Medizin und Chirurgie des Rindes 2002 Berlin, Parey Buchverlag 469 473

[B4] Radostits OM Gay CC Hinchcliff KW Constable PD Impaction of the omasum Veterinary Medicine. A Textbook of the Diseases of Cattle, Horses, Sheep, Pigs and Goats 2007 Philadelphia, Saunders Elsevier 352 353

[B5] Cegarra IJ Lewis RE Contrast study of the gastrointestinal tract in the goat (Capra hircus) Am J Vet Res 1977 38 1121 1128 911078

[B6] Irmer M Computertomographische Untersuchung des Abdomens bei 30 Ziegen Dr Med Vet Thesis 2010 University of Zurich

[B7] Braun U Blessing S Ultrasonographic examination of the omasum in 30 healthy cows Vet Rec 2006 159 812 815 10.1136/vr.159.22.750 17158713

[B8] Braun U Blessing S Lejeune B Hässig M Ultrasonography of the omasum in cows with various gastrointestinal diseases Vet Rec 2007 160 865 869 10.1136/vr.160.25.865 17586790

[B9] Braun U Irmer M Steininger K Schade B Ultraschallbefunde bei einer Ziege mit Aszites infolge Mesotheliom Schweiz Arch Tierheilk 2009 151 397 400 10.1024/0036-7281.151.8.397 19653164

[B10] Becker-Birck M Computertomographische Untersuchung des Thorax bei 30 Ziegen Dr Med Vet Thesis 2009 University of Zurich

[B11] Jacquat D Sonographische Untersuchung von Haube, Pansen, Psalter und Labmagen bei 30 Ziegen Dr Med Vet Thesis 2010 University of Zurich

[B12] Steininger K Ultraschalluntersuchung von Leber, Milz, Dünndarm, Dickdarm und Harnapparat bei 30 Ziegen Dr Med Vet Thesis 2010 University of Zurich

[B13] Braun U Sicher D Pusterla N Ultrasonography of the lungs, pleura, and mediastinum in healthy cows Am J Vet Res 1996 57 432 438 8712503

[B14] Braun U Ultrasonography of the gastrointestinal tract in cattle Vet Clin North Am (Food Anim Pract) 2009 25 567 590 10.1016/j.cvfa.2009.07.004 19825434

[B15] Mohindroo J Kumar A Sangwan V Udehiya R Singh SS Ultrasonographic evaluation of the omasum in cows and buffaloes Vet Radiol Ultrasound 2008 49 295 299 10.1111/j.1740-8261.2008.00368.x 18546788

